# Translation, Cultural Adaptation, and Validation of the Arab Version of the Adolescent Food Parenting Questionnaire (AFPQ) in Kut City, Iraq

**DOI:** 10.7759/cureus.70739

**Published:** 2024-10-02

**Authors:** Abbas Ali Alkinani, A J Rohana, Ruhaya Hasan, Wan Muhamad Amir W Ahmad, Saad Abid Al-Badri

**Affiliations:** 1 Department of Community Medicine, School of Medical Sciences, Universiti Sains Malaysia, Kubang Kerian, MYS; 2 School of Dental Sciences, Universiti Sains Malaysia, Kubang Kerian, MYS; 3 Department of Internal Medicine, College of Medicine, Wasit University, Wasit, IRQ

**Keywords:** adolescent, adolescent food parenting behaviors, arabic translation, cultural adaptation, translation, validation

## Abstract

Background

Food parenting behaviors have been increasingly critical to adolescent nutritional health. These behaviors play a decisive role in shaping food intake and weight status in adolescents through autonomy support, coercive control, modeling habits, healthy structure, and determining snacking patterns. To our knowledge, there is no available food parenting questionnaire in the Arabic version; therefore, a reliable and validated Arabic version of the questionnaire to assess food parenting behavior among Iraqi adolescents is required. The aim of this study was to translate, culturally adapt, and validate the Adolescent Food Parenting Questionnaire (AFPQ) into Arabic (AFPQ-A) for adolescents aged 16 to 18 in Kut City, Iraq.

Methodology

The original version of AFPQ was translated according to a 10-step protocol after being authorized by the developer. The modified kappa statistic (κ_m_), the sum content validity index/universal agreement (S-CVI/UA) method, the scale-level content validity index average (S-CVI/Ave), and the item-level content validity index (I-CVI) were used by four independent experts to assess the content validity of the AFPQ for adolescents and parenting. Face validity was assessed among the adolescents (n = 30) and their respective parents.

Results

The translation process into the Arabic version produced minor modifications to the original questionnaire. The S-CVI/Ave score for the parent version was 0.95, while the I-CVI/UA score was 0.81 for the adolescent version. The κ_m_ values of 1.00 were found in the majority of the items. These scores indicated that the AFPQ items possessed good content validity. Both the parent and adolescent versions demonstrated excellent face validity, with the sum of face validity index/average (S-FVI) scores of 0.95 and 0.94, respectively.

Conclusion

The AFPQ-A has been demonstrated to be a valid and reliable instrument for the assessment of food parenting behaviors in Iraqi adolescents.

## Introduction

Food parenting behaviors are increasingly recognized as critical to the nutritional and health status of adolescents [1,2]. Adolescent food parenting (AFP) is characterized by five essential domains: autonomous support, healthy structure, coercive control, snack structure, and modeling, each of which plays a pivotal role during the developmental period of adolescence [3]. Food parenting practices (FPPs) refer to context-specific parental behaviors associated with food and eating, designed to influence the socialization of children and adolescents toward particular dietary habits and behaviors [1]. The World Health Organization (WHO) identifies adolescence, spanning from ages 10 to 18, as a critical phase marked by significant physical and cognitive growth, during which habits and lifestyle patterns are established and remain adaptable [4].

The concept of food-parent behaviors represents an innovative framework that examines the interaction between adolescents and their parents, particularly in terms of autonomy support, coercive control, modeling, healthy structure, and snack structure, all of which contribute to healthy adolescent development [1,3]. Historically, these behaviors have been explored independently in either parents or adolescents. However, emerging research highlights the importance of a more holistic understanding of FPPs [3]. Despite this, little is known about the importance of AFP behaviors for healthy development and their impact on food intake and weight status, specifically among the Iraqi communities.

The cultural context and community environment within Arabic societies play a pivotal role in shaping parental practices related to food. For instance, research conducted in Saudi Arabia highlighted that numerous mothers demonstrated limited awareness of proper food storage and utilization, insufficient understanding of personal hygiene and foodborne diseases, as well as inadequate knowledge of correct food handling procedures [5]. Another study examined parent-teen interactions about food and found a relationship between reported food practices by parents and disordered eating in their adolescents. Apart from that, parent-teen interactions during food-related disagreements were observed [6]. In managing fruit and vegetable consumption for children at home, various handling methods were influenced by demographics, diet, and parental authority [7]. Additionally, FPPs during independent adolescent eating revealed common components between parents and adolescents [8]. Apart from that, different feeding patterns in junk food and sugary drinks were associated with varying consumption levels in both parents and children [7]. Apart from junk food and sweetened sugar-sweetened beverages (SSBs), street food vendors that do not comply with hygienic standards were also the main factors influencing eating behavior among Iraqi adolescents [9]. Research investigating food parenting behavior is still scarce, especially among Iraqi adolescents. Therefore, developing a suitable instrument based on diverse cultures and languages is necessary. It requires more literal translation; it necessitates cross-cultural translation and validation [9]. Arabic, Iraq's national and official language, is widely spoken across various dialects [10].

In order to ensure that a translated measurement scale accurately reflects the original, a meticulous translation process is essential [11]. The AFPQ, developed by Koning et al. (2021) [3], is a validated tool for assessing AFP behaviors. This research aims to address the gaps by translating, culturally adapting, and validating the Adolescent Food Parenting Questionnaire Arabic Version (AFPQ-A) for use with families in Iraq.

## Materials and methods

The cultural translation and adaptation of the AFPQ

The AFPQ has been established as a comprehensive instrument designed to assess FPPs from both parental and adolescent perspectives. This dual-assessment approach utilized separate questionnaires for parents and adolescents, enabling the examination of the relationship between parental practices and dietary behaviors in their adolescents. Developed by Koning and colleagues, the AFPQ evaluated FPP across five key domains: autonomy support, coercive control, modeling, healthy structure, and snack structure, providing valuable insights into how these practices influence dietary habits in adolescents [3].

The AFPQ consists of 16 questions, with each question corresponding to one of the five domains. The autonomy support domain includes questions 1, 5, and 9; the coercive control domain comprises questions 4, 7, 10, and 14; modeling is addressed by questions 11 and 15; the healthy structure is covered by questions 3 and 6; and the snack structure domain includes questions 8, 12, 13, and 16. Both parents and adolescents rated each item on a five-point Likert scale, where one indicates strongly disagree, and five indicates strongly agree, based on Koning and colleagues [3]. Cronbach's alphas for these five factors range from 0.74 to 0.85, indicating high reliability.

**Figure 1:**
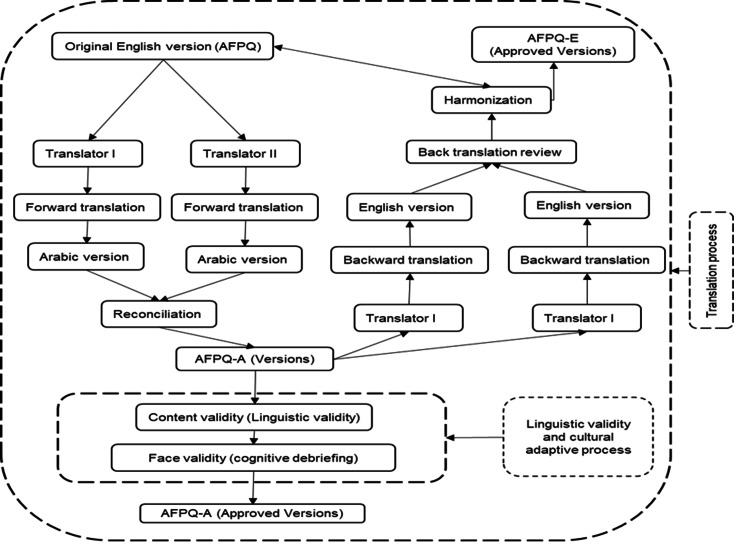
The flowchart of the culturally adapted translation of the Arabic version of AFPQ AFPQ = Adolescent Food Parenting Questionnaire, AFPQ-A = Adolescent Food Parenting Questionnaire Arabic Version, and AFPQ-E = Adolescent Food Parenting Questionnaire English Version

The developer for the AFPQ granted approval and permission to translate and validate the questionnaire into Arabic versions and use it among Iraqi adolescents [3]. Over the past seven decades, numerous relationships have been identified between parental behaviors and the psychological, behavioral, and physiological development of their respective children [12]. In the present study, the translation process adhered to the specific guidelines [13], as shown in Figure 1.

1. Preparation Step: A literature review was conducted to identify an appropriate questionnaire for assessing parental feeding food-feeding practices and their relationship with the nutritional status of their respective children/adolescent adolescents. After a few considerations, eventually, the AFPQ was selected. Apart from the developer’s permission and approval, a literature review was conducted to identify a validated questionnaire suitable for translation.

2. Forward Translation Step: Two native Iraqis who were proficient in both English and Arabic independently translated the questionnaires from English to Arabic (draft 1). One of the translators holds a Master's degree in Linguistics and Translation, while the other is pursuing a PhD program in public health. Each translation was later reviewed and summarized for easy reference. Upon finalizing the translated scale, approval was granted by the project coordinator.

3. Reconciliation Step: A panel meticulously reviewed each forward translation, diligently understood thoroughly, and reconciliation was conducted if any discrepancies existed through comprehensive discussions to ensure cultural appropriateness. Subsequently, a standardized forward translation of the AFPQ-A was meticulously developed and formally approved by the review committee.

4. Backward Translation Step: The research team performed back translation on the Arabic versions of AFPQ to ensure compatibility and accuracy. This process involved independent translators translating the Arabic version back into English without prior knowledge of the original questionnaires or the study's purpose. The translators were proficient in both English and Arabic, facilitating effective communication. The translation team consisted of a medical professional, currently a PhD candidate pursuing a family medicine program at the Universiti Sains Malaysia, and an English language instructor from the Iraqi Ministry of Education. Figure 1 outlines the translation and validation steps for AFPQ into Arabic. The research team confirmed conceptual, item, and semantic equivalence between the English and Arabic versions through this rigorous translation process of the draft 2 version [14].

5. Backward Translation Review Step: The expertise team, responsible for both forward and backward translation reviews, confirmed conceptual, item, and semantic equivalence [14]. Conceptual equivalence ensured the presence and acceptability of adolescent parent feeding practices. Item equivalence focuses on the relevance and acceptability of individual items within their designated groups [15]. Semantic compatibility between the English and Arabic versions of the draft 2 version of the AFPQ was ensured through rigorous forward and backward translation processes.

6. Harmonization Step: Differences between forward (draft 1) and backward translations (draft 2) were identified and resolved to ensure conceptual equivalence with the original instrument. The review committee confirmed that the common forward translation of AFPQ-A (draft 1) matched the English version of AFPQ, establishing it as the pre-final version for testing (pre-final of AFPQ-A).

7. Cognitive Debriefing Step: The pre-final AFPQ-A underwent cognitive debriefing with 10 secondary school students aged 16-18 and their parents in Kut district. Participants were briefed on the purpose and process of debriefing. Each participant received a pre-final AFPQ-A and provided feedback on clarity, comprehension, and cultural relevance.

8. Review of Cognitive Debriefing Results and Finalization Step: Feedback from the cognitive debriefing sessions was integrated to refine the translation. The existence of inconsistencies between respondents' interpretations and the original text was identified and addressed based on committee consensus.

9. Proofreading Step: The final translation was proofread to ensure accuracy and consistency. This step aimed to detect and correct any remaining errors before the questionnaire was administered to the target population.

10. Production of Final Arabic Version Step: A comprehensive report documenting the translation process was prepared, detailing all decisions made to ensure consistency for future translations of the same measure. The entire process is illustrated in Figure 1: flowchart of translating, culturally adapting, and validating the AFPQ.

Validation process of the AFPQ

Content Validity (Linguistic Validation)

The content validation process began with the appointment of four independent experts from Iraq who were fluent in both Arabic and English. This panel validated the content of the APFQ-A, adhering to the acceptable standard [16,17].

The expert committee included a lecturer from the Department of Translation, College of Arts, Wasit University in Iraq, and two nutritionists from the University of Putra Malaysia. All were native Arabic speakers with strong proficiency in English. They reviewed the validity methods, word appropriateness, and content to ensure cultural relevance for the Iraqi population.

The content validity of the final version of the questionnaire was evaluated using established content validity methods, including the item-level content validity index (I-CVI), scale-level content validity index average (S-CVI/Ave), the sum content validity index/universal agreement (S-CVI/UA) method, and the modified kappa statistic. These indices assess the level of agreement among experts in rating the relevance of each item [18]. A consensus was achieved regarding the relevance, clarity, simplicity, and absence of ambiguity for all items in the AFPQ-A, as well as the definitions associated with each item [9,18]. Experts evaluated the scale items using a four-point ordinal rating system (1 = not relevant, 2 = needs revision, 3 = relevant but requires minor revision, 4 = highly relevant). Items receiving a rating of one or two were deemed invalid, while those rated three or four were considered valid, in alignment with conceptual definitions [9].

The content validity index (CVI) is assessed at both the I-CVI and the S-CVI [18]. The I-CVI measures the proportion of agreement on individual items, with values ranging from zero to one, while the S-CVI reflects the proportion of items rated as content valid at the scale level. Using the S-CVI/UA, the S-CVI indicates the percentage of items that receive unanimous agreement from raters [18]. Additionally, the kappa statistic accounts for chance agreement, offering an adjusted measure of inter-rater reliability.

The formulas employed for assessing item-specific content validity indices are delineated below [16,18-21]. An item is approved if I-CVI ≥ 0.78 [16,17]. According to Polit, Beck, and Owen (2007), instruments with an agreement rate of 80% or greater should be prioritized. Items with I-CVI ≥ 0.79 are considered appropriate; those between 0.70 and 0.79 need revision, and those ≤ 0.70 are removed. An S-CVI/Ave of ≥0.90 indicates acceptable content validity [16,22]. S-CVI/UA values of 0.70 indicate that, on average, 70% of the items were unanimously agreed upon by experts as relevant [16,22].

Lastly, the kappa statistic evaluates the degree of agreement beyond what would be expected by chance. Values below zero signify less than chance agreement, while values ranging from 0.01 to 0.20 indicate slight agreement. Scores between 0.21 and 0.40 reflect fair agreement, those from 0.41 to 0.60 denote moderate agreement, and values within the range of 0.61 to 0.80 represent good agreement. Scores from 0.81 to 0.99 suggest almost perfect agreement [23].

Formulas: 

$I-CVI = (number of experts rating item as 3 or 4) / (total number of experts)$

where I denotes an item, and CVI refers to the content validity index [16].

$SCVI/Ave = (\Sigma I-CVI) / N$

where S represents the scale, CVI denotes the content validity index, Σ signifies the sum, and N indicates the total number of items [19].

 $𝑆&minus;𝐶𝑉𝐼/𝑈𝐴 = 𝑚 / 𝑁$

where S denotes the scale, CVI represents the content validity index, UA signifies universal agreement, 𝑚 refers to the number of items with unanimous agreement, and N indicates the total number of items [19].

 $𝑃𝑐 = (𝑁! / (𝐴! &lowast; (𝑁 &minus; 𝐴)!)) &lowast; 5^𝑁$

where *Pc* represents the probability of chance agreement, N denotes the number of experts, A signifies the number of items agreed upon, ! indicates the factorial, and 5 refers to the number of rating categories [18,21].

$&kappa; = (I-CVI &minus; 𝑃𝑐) / (1 &minus; 𝑃𝑐)$

where κ denotes kappa, I represents an item, CVI stands for content validity index, and *Pc* refers to the probability of chance agreement [20].

Face Validity

Face validity refers to the degree to which the content of items aligns with the construct definition, as determined solely by the researcher's subjective judgment [24]. Once content validity is established, the validity of the response process is evaluated [18]. The concept of "face validity" specifically denotes the extent to which evaluators perceive the questions in an assessment tool as being relevant to the targeted construct and objectives.

Adolescents and their parents were the intended recipients of an assessment instrument. Although it was a general practice that 10 raters were the minimum number for validating a response process, the previous research used 30 raters [25]. According to previous studies, 30 raters were considered the highest and most acceptable to conduct face validity [26]. In the present study, face-to-face validation of the response procedure was carried out among 30 students from four Kut City schools (15 boys and 15 girls). Raters were solicited for feedback both verbally and in writing for improvement such as ensuring clarity and understandability of the items. Every item was evaluated by each rater on a scale from one to four to distinguish between clear (three or four) and unclear (one or two) items. Multiple studies have established a minimum acceptable Face Validity Index (FVI) score of at least 0.80, a criterion pivotal to this study's assessment accuracy [26]. The FVI was calculated using specific indices: item face validation index (I-FVI) = (number of agreed items) / (total number of raters), and the sum of face validity index/average (S-FVI) = (sum of I-FVI scores) / (number of items) [18].

Ethical considerations

The ethical clearance for the study protocol (code: USM/JEPeM/KK/23030276) was obtained from the Human Research Ethics Committee at the Universiti Sains Malaysia. Additionally, ethical approval for human subject participation was granted by the Branch of Studies and Research, Section of Training and Preparation, under the Director General of Wasit Education (reference no. 71654, dated November 28, 2022).

## Results

Cultural translation and adaptation of the AFPQ

Several modifications were made to both the parent and adolescent versions of the AFPQ during the translation process to enhance clarity and cultural relevance. For item Q4, examples like giving a piece of fruit while studying were added to help children relax and focus better. In item Q6, “easily” replaced "quickly” to emphasize effortless vegetable consumption, with examples such as having salad with the main meal to illustrate daily routines. Item Q7 included examples like “a piece of cake after completing school assignments” to clarify how rewards were applied, enhancing understanding of parenting practices. For Q11, examples, such as having fresh salad or seasonal fruits during dinner, were used to signify healthy eating habits. Item Q14 justified “a small snack, like a bite-sized treat” to convey comfort through food, resonating culturally. These modifications, implemented in both forward and backward translations, ensure consistency and comprehensibility for both parents and adolescents. Appendix Table 3 provides a detailed account of the translation and back-translation procedures employed for the AFPQ. This table outlines the systematic approach undertaken to ensure the linguistic and conceptual accuracy of the AFPQ through both the translation and back-translation phases.

The evaluation of the expert committee led to minor revisions in both versions of the AFPQ. For example, in item Q2, “having healthy meals together and avoiding unhealthy foods” was suggested to enhance clarity and communication effectiveness, illustrating how eating behavior was applied in daily life. Paraphrasing item Q3a to “I always have fruits and veggies to eat at home” was simplified and clarified based on the original statement about the consistent availability and consumption of fruits and vegetables.

Content validity

According to the feedback from the experts on content validation, the AFPQ-A was relevant to its utilization, high clarity, simplicity, and clarity. The remaining AFPQ-A items had scale-level content validity index average (S-CVI/Ave) and S-CVI/UA scores of 0.95 and 0.81, respectively. However, six items, Q2p, Q2a, Q3a, Q9p, Q9a, and Q14p, needed revision as their I-CVI scores for relevance, clarity, simplicity, and ambiguity were below 0.75. Expert opinions and recommendations from the panel and committee guided the revisions to these items. The I-CVI revealed that most items were deemed suitable in terms of relevance, clarity, simplicity, and lack of ambiguity, as presented in Table 1. Notably, the majority of AFPQ-A items demonstrated high inter-rater agreement, indicated by an excellent modified kappa coefficient (κ = 1.0). However, specific items, Q2p, Q2a, Q3a, Q9p, Q9a, and Q14p, exhibited moderately adjusted kappa values (κ = 0.67) across these same domains of evaluation.

**Table 1:** Expert agreement (n = 4) on the content validity of items in the Arabic version of the AFPQ App. = approved, a = adolescent version, EC = expert consensus, Exc. = excellent, Gd. = good, I-CVI = item-level content validity index, κ_m_ = modified kappa agreement, p = parent version, Rc = probability of chance occurrence, Rev. = need revision, S-CVI/Ave = scale-level content validity index average (number of items), S-CVI/UA = sum content validity index/universal agreement

Item	Relevance				Clarity				Simplicity				Ambiguity				Interpretation	
	EC	I-CVI	Rc	κ_m_	EC	I-CVI	Rc	κ_m_	EC	I-CVI	Rc	κ_m_	EC	I-CVI	Rc	κ_m_	I-CVI	κ_m_
Q1_p_	4	1	0.06	1.00	4	1	0.06	1.00	4	1	0.06	1.00	4	1	0.06	1.00	App.	Exc.
Q1_a_	4	1	0.06	1.00	4	1	0.06	1.00	4	1	0.06	1.00	4	1	0.06	1.00	App.	Exc.
Q2_p_	3	0.75	0.25	0.67	3	0.75	0.25	0.67	3	0.75	0.25	0.67	3	0.75	0.25	0.67	Rev.	Gd.
Q2_a_	3	0.75	0.25	0.67	3	0.75	0.25	0.67	3	0.75	0.25	0.67	3	0.75	0.25	0.67	Rev.	Gd.
Q3_p_	4	1	0.06	1.00	4	1	0.06	1.00	4	1	0.06	1.00	4	1	0.06	1.00	App.	Exc.
Q3_a_	3	0.75	0.25	0.67	3	0.75	0.25	0.67	3	0.75	0.25	0.67	3	0.75	0.25	0.67	Rev.	Gd.
Q4_p_	4	1	0.06	1.00	4	1	0.06	1.00	4	1	0.06	1.00	4	1	0.06	1.00	App.	Exc.
Q4_a_	4	1	0.06	1.00	4	1	0.06	1.00	4	1	0.06	1.00	4	1	0.06	1.00	App.	Exc.
Q5_p_	4	1	0.06	1.00	4	1	0.06	1.00	4	1	0.06	1.00	4	1	0.06	1.00	App.	Exc.
Q5_a_	4	1	0.06	1.00	4	1	0.06	1.00	4	1	0.06	1.00	4	1	0.06	1.00	App.	Exc.
Q6_p_	4	1	0.06	1.00	4	1	0.06	1.00	4	1	0.06	1.00	4	1	0.06	1.00	App.	Exc.
Q6_a_	4	1	0.06	1.00	4	1	0.06	1.00	4	1	0.06	1.00	4	1	0.06	1.00	App.	Exc.
Q7_p_	4	1	0.06	1.00	4	1	0.06	1.00	4	1	0.06	1.00	4	1	0.06	1.00	App.	Exc.
Q7_a_	4	1	0.06	1.00	4	1	0.06	1.00	4	1	0.06	1.00	4	1	0.06	1.00	App.	Exc.
Q8_p_	4	1	0.06	1.00	4	1	0.06	1.00	4	1	0.06	1.00	4	1	0.06	1.00	App.	Exc.
Q8_a_	4	1	0.06	1.00	4	1	0.06	1.00	4	1	0.06	1.00	4	1	0.06	1.00	App.	Exc.
Q9_p_	3	0.75	0.25	0.67	3	0.75	0.25	0.67	3	0.75	0.25	0.67	3	0.75	0.25	0.67	Rev.	Gd.
Q9_a_	3	0.75	0.25	0.67	3	0.75	0.25	0.67	3	0.75	0.25	0.67	3	0.75	0.25	0.67	Rev.	Gd.
Q10_p_	4	1	0.06	1.00	4	1	0.06	1.00	4	1	0.06	1.00	4	1	0.06	1.00	App.	Exc.
Q10_a_	4	1	0.06	1.00	4	1	0.06	1.00	4	1	0.06	1.00	4	1	0.06	1.00	App.	Exc.
Q11_p_	4	1	0.06	1.00	4	1	0.06	1.00	4	1	0.06	1.00	4	1	0.06	1.00	App.	Exc.
Q11_a_	4	1	0.06	1.00	4	1	0.06	1.00	4	1	0.06	1.00	4	1	0.06	1.00	App.	Exc.
Q12_p_	4	1	0.06	1.00	4	1	0.06	1.00	4	1	0.06	1.00	4	1	0.06	1.00	App.	Exc.
Q12_a_	4	1	0.06	1.00	4	1	0.06	1.00	4	1	0.06	1.00	4	1	0.06	1.00	App.	Exc.
Q13_p_	4	1	0.06	1.00	4	1	0.06	1.00	4	1	0.06	1.00	4	1	0.06	1.00	App.	Exc.
Q13_a_	4	1	0.06	1.00	4	1	0.06	1.00	4	1	0.06	1.00	4	1	0.06	1.00	App.	Exc.
Q14_p_	3	0.75	0.25	0.67	3	0.75	0.25	0.67	3	0.75	0.25	0.67	3	0.75	0.25	0.67	Rev.	Gd.
Q14_a_	4	1	0.06	1.00	4	1	0.06	1.00	4	1	0.06	1.00	4	1	0.06	1.00	App.	Exc.
Q15_p_	4	1	0.06	1.00	4	1	0.06	1.00	4	1	0.06	1.00	4	1	0.06	1.00	App.	Exc.
Q15_a_	4	1	0.06	1.00	4	1	0.06	1.00	4	1	0.06	1.00	4	1	0.06	1.00	App.	Exc.
Q16_p_	4	1	0.06	1.00	4	1	0.06	1.00	4	1	0.06	1.00	4	1	0.06	1.00	App.	Exc.
Q16_a_	4	1	0.06	1.00	4	1	0.06	1.00	4	1	0.06	1.00	4	1	0.06	1.00	App.	Exc.
S-CVI/Ave_(p&a)_	-	0.95	-	-	-	0.95	-	-	-	0.95	-	-	-	0.95	-	-	-	-
S-CVI/UA_(p&a)_	-	0.81	-	-	-	0.81	-	-	-	0.81	-	-	-	0.81	-	-	-	-

Face validation

The FVI of the AFPQ-A for parents and adolescents was S-FVI/Ave = 0.95 and 0.94, respectively, indicating robust consensus among raters on the clarity and comprehensibility of the items, in short demonstrating a satisfactory and adequate level of face validity. Table 2 summarizes the item-specific face validity indices (I-FVI range from 0.80 to 1.00), confirming their approval.

**Table 2:** The average item for the face validity index of AFPQ-A from 30 raters App. = approved, I-FVI = item face validation index, INTP = interpretation, S-FVI/Ave = sum of face validity index/average (number of items)

Parent				Adolescent			
Item	Raters in agreement	I-FVI	INTP	Item	Raters in agreement	I-FVI	INTP
Q1	30	1.00	App.	Q1	30	1.00	App.
Q2	24	0.80	App.	Q2	24	0.80	App.
Q3	29	0.97	App.	Q3	30	1.00	App.
Q4	24	0.80	App.	Q4	24	0.80	App.
Q5	30	1.00	App.	Q5	30	1.00	App.
Q6	29	0.97	App.	Q6	27	0.90	App.
Q7	30	1.00	App.	Q7	29	0.97	App.
Q8	30	1.00	App.	Q8	30	1.00	App.
Q9	30	1.00	App.	Q9	30	1.00	App.
Q10	30	1.00	App.	Q10	30	1.00	App.
Q11	26	0.87	App.	Q11	25	0.83	App.
Q12	27	0.90	App.	Q12	25	0.83	App.
Q13	30	1.00	App.	Q13	30	1.00	App.
Q14	30	1.00	App.	Q14	29	0.97	App.
Q15	27	0.90	App.	Q15	26	0.87	App.
Q16	30	1.00	App.	Q16	30	1.00	App.
S-FVI/Ave		0.95		S-FVI/Ave		0.94	

## Discussion

Based on the recommendations [13] and reviewed by an expert panel, the translation procedure for the present study closely adheres to those guidelines. Following this process ensures that the translated questionnaire adheres to the protocol based on the abovementioned guidelines and that the translated items remain close to the original version of the questionnaire. To our knowledge, this was the first study to translate and adapt a measure of AFP practices in Iraq. The study evaluated the content and face validity of the AFPQ-A in Arabic, resolving minor translation discrepancies to produce the final Arabic versions of the questionnaire. Arabic is the fifth most spoken language in the world, with more than 450 million native speakers. On the other hand, 22 nations recognized it as an official language [27]. The process of cross-cultural adaptation involved not only translation but also culturally appropriate amendments for a new context [28]. Despite extensive geographical diversity in Iraq, the Arabic language exhibits a wide range of accents, slang, and subtleties. During this research, challenges in finding accurate Arabic equivalents for terms like “a small snack as comfort” during the reconciliation process were identified. Consequently, both the Arabic term “كالتعتيمة” and the English term “such as a bite-sized treat” in item Q14 were consented to by the panel committee and the researcher. Such translation efforts often encounter difficulties when languages lack direct equivalents, potentially resulting in divergent interpretations [28]. This highlights the growing importance of cross-cultural research translation within the field of health research. Additionally, items that posed challenges, such as those requiring clarification with illustrative examples, were modified based on alternative suggestions from the panel committee, leading to a reorganization of Arabic terminology aimed at enhancing clarity.

The majority of the questions in the cognitive interviews were comprehended by the parents and the adolescents, although they did suggest changes to the questionnaire's structure, wording, and example usage. Additionally, both participants outlined the importance of adding examples to the corrosive control-related items Q7p and Q7a. These modifications emphasized the necessity of carrying out cognitive interview research prior to the use of newly developed or modified measurement instruments in diverse cultural contexts.

The key finding of the present study was the complexity and confusion of the items. There was an effort to refine the sentence structure and provide more context in items 2, 4, 6, and 11. These terms were likewise deemed unclear by experts. Examples such as “having healthy meals together and avoiding unhealthy foods,” “giving them a piece of fruit while they are studying to help them rest and focus better,” “when they have salad served with the main meal,” and “having a fresh salad or seasonal fruits during dinner” was provided to minimize the obscurity. This study emphasized the significance of providing clear and simple assessment items, as well as adjusting them before utilizing them in research settings, particularly among parents with poor levels of education and reading skills.

This study confirms that the AFPQ-A has good, clear, simple, and essential content validity based on relevance, clarity, simplicity, and ambiguity. In terms of assessment, accuracy, and suitability in translation items provided a holistic perspective of content validity.

The translated version must be culturally adapted and exhibit an acceptable level of content validity, as indicated by an I-CVI or an average I-CVI (I-CVI/Ave) of ≥0.90 [22] or ≥0.80 [18]. Moreover, it should maintain the same communicative intent as the original instrument. In this study, the AFPQ-A demonstrated strong content validity for the Iraqi population, achieving an I-CVI/Ave of 0.95 and an I-CVI for universal agreement (I-CVI/UA) of 0.81. The kappa statistic revealed that the majority of items had values of 1.00, significantly supporting the CVI results and indicating a level of agreement beyond chance. To improve clarity and eliminate ambiguity, items Q2p, Q2a, Q3a, Q9p, Q9a, and Q14p were revised.

Face validation verified that respondents understood the questions. The final Arabic version questionnaire was simple, easy to read, and uncomplicated, producing good responses from the respondents. The FVI for clarity and comprehension of the current questionnaire among parents and their adolescents ranged from 0.80 to 1.00 (I-FVI), with an S-FVI/Ave of 0.95 and 0.94, respectively. Many researchers recommended that an I-FVI value above 0.80 and an S-FVI/Ave value above 0.90 were acceptable for inter-rater agreement in questionnaires [26]. Nonetheless, further assessment is required to confirm its construct validity.

There were a few study limitations. Language and cultural adaptation were common issues in the English-to-Arabic translation [29]. A few English terms were mistranslated into Arabic, leading to different meanings for the same concept. Additionally, some Arabic terminology was not suitable for translation.

Eventually, the findings from the present study recommended the use of the AFPQ-A to measure AFP behaviors in Iraqi adolescents despite the limitations highlighted earlier. The AFPQ-A offers additional advantages for the clinical evaluation of adolescent obesity, including reduced burden costs, applicability to a wide sample population, and rapid assessment in nearby facilities. The study suggested further investigation into the relationships, development, and associated health effects in young children. Childhood obesity and food parenting have been well-known public health issues linked to numerous negative health outcomes.

Nutrition-related studies will improve public health, especially in Iraq, by addressing the rising epidemics of childhood obesity and nutritional disorders. Healthy eating habits among adolescents were associated with better health outcomes, including increasing parental support for autonomy and health structure, while unhealthy eating habits were linked to less coercive control and poor snack structure [3]. The present study has the potential to be a useful indicator of food parenting behaviors, provide evidence of risks, and facilitate earlier population-level intervention through more relevant and focused programs, given its primary focus on young children.

## Conclusions

The results demonstrated that the AFPQ-A possesses acceptable content and face validity, establishing it as a reliable tool for measuring food-related parenting practices among Iraqi adolescents in Wasit. By employing this validated tool, researchers can gain insights into these behaviors and develop more effective early intervention programs aimed at improving food-related outcomes in this population, especially in Arab countries.

## Appendices

**Table 3:** Translation and back translation of the AFPQ A = Arabic version item, AS = autonomy support (دعم الاستقلال), B = back-translated English version item, CC = coercive control (التحكم القسري), HS = healthy structure (الهيكل الصحي), Mod = modeling (النمذجة), O = original English version item, SS = snack structure (هيكل الوجبات الخفيفة)

Item no.	Parent version	Adolescent version	Domain
01	(O) I educate my child about nutrition, for example, talking about healthy and unhealthy food. (A) انا أقوم بتثقيف طفلي حول التغذية، على سبيل المثال الحديث عن الطعام الصحي وغير الصحي. (B) I educate my child about nutrition, for example, talking about healthy and unhealthy food.	(O) My parents educate me about nutrition, for example, talking about healthy and unhealthy food. (A) يعلمني والداي عن التغذية، على سبيل المثال الحديث عن الطعام الصحي وغير الصحي. (B) My parents educate me about nutrition, for example talking about healthy and unhealthy food.	AS
02	(O) I explain why I have specific rules about eating to my child. (A) انا أشرح لطفلي لماذا لدي قواعد معينة بشأن تناول الطعام، مثل تناولنا وجبات صحية معًا وتجنبنا الأطعمة غير الصحية. (B) I explain to my child why I have specific rules about eating, such as having healthy meals together and avoiding unhealthy foods.	(O) My parents explain why they have specific rules about eating to me. (A) يشرح لي والداي سبب وجود قواعد معينة حول تناول الطعام ، مثل تناول وجبات صحية معًا وتجنب الأطعمة غير الصحية. (B) My parents explain to me why they have specific rules about eating, such as having healthy meals together and avoiding unhealthy foods	AS
03	(O) There are always fruit and vegetables at home for my children to eat. (A) هناك دائمًا فواكه وخضروات في المنزل ليأكلها أطفالي. (B) There are always fruit and vegetables at home for my children to eat.	(O) There are always fruit and vegetables at home for me to eat. (A) لدي دائمًا فواكه وخضروات لأتناولها في المنزل. (B) I always have fruits and veggies to eat at home.	HS
04	(O) I sometimes give my child something to eat as a distraction. (A) أحيانًا أعطي طفلي شيئًا ليأكله لإلهائه ، مثل إعطائه قطعة من الفاكهة أثناء دراسته ليستريح ويركز بشكل أفضل. (B) Sometimes I give my child something to eat to distract them, like giving them a piece of fruit while they are studying to help them rest and focus better.	(O) My parents sometimes give me something to eat as a distraction. (A) أحيانًا يعطيني والدي شيئًا لآكله لإلهائي، مثل إعطائي قطعة من الفاكهة أثناء دراستي لأستريح وأركز بشكل أفضل. (B) Sometimes my parent gives me something to eat to distract me, like giving me a piece of fruit while I am studying to help me rest and focus better.	CC
05	(O) I give my child feedback related to their eating habits, for example, if my child eats too quickly or doesn’t eat enough vegetables. (A) انا أقدم لطفلي ملاحظات تتعلق بعاداته الغذائية، على سبيل المثال إذا كان طفلي يأكل بسرعة كبيرة أو لا يأكل ما يكفي من الخضار. (B) I give my child feedback related to their eating habits. For example, if my child eats too quickly or doesn’t eat enough vegetables.	(O) My parents give me feedback related to my eating habits, for example, if I eat too quickly or don’t eat enough vegetables. (A) يقدم لي والداي ملاحظات تتعلق بعاداتي في الأكل ، على سبيل المثال إذا أكلت بسرعة كبيرة أو لم أتناول ما يكفي من الخضار. (B) My parents gave me feedback related to my eating habits. For example, if I eat too quickly or don’t eat enough vegetables.	AS
06	(O) At home, my child can quickly eat vegetables as they are part of our daily meals. (A) في المنزل, يمكن لطفلي أن يأكل الخضروات بسهولة لأنها جزء من وجباتنا اليومية ، مثل تناوله للسلطة التي تُقدم مع الوجبة الرئيسية. (B) At home, my child can easily eat vegetables because they are part of our daily meals, like when they have salad served with the main meal.	(O) At home, I can quickly eat vegetables as they are part of our daily meals. (A) في المنزل يمكنني أكل الخضروات بسهولة لأنها جزء من وجباتنا اليومية، مثل السلطة التي تقدم مع كل وجبة رئيسية. (B) At home, I can easily eat vegetables because they are part of our daily meals, like the salad served with every main meal.	HS
07	(O) I sometimes give my child something to eat as a reward. (A) أحيانًا أعطي طفلي شيئًا ليأكله كمكافأة، مثل قطعة من الكعك بعد إنهاء واجباته المدرسية. (B) Sometimes I give my child something to eat as a reward, like a piece of cake after he / his finishes his school assignments.	(O) My parents sometimes give me something to eat as a reward. يعطيني والداي أحيانًا شيئًا ما آكله كمكافأة، مثل قطعة من الكعك بعد إنهاء واجباتي المدرسية. (B) My parents sometimes give me something to eat as a reward, like a piece of cake after finishing my school assignments.	CC
08	(O) I let my children snack if they want to. (A) أنا أسمح لطفلي بتناول وجبة خفيفة إذا أراد ذلك. (B) I let my child snack if they want to.	(O) My parents let me snack if I want to. (A) سمح لي والداي بوجبة خفيفة إذا أردت ذلك. (B) My parents let me snack if I want to.	SS
09	(O) I discuss why it is important to eat fruit and vegetables with my child. (A) انا أناقش مع طفلي سبب أهمية تناول الفاكهة والخضروات. (B) I discuss with my child why it is important to eat fruit and vegetables.	(O) My parents discuss why it is important to eat fruit and vegetables with me. (A) يناقش والداي معي سبب أهمية تناول الفاكهة والخضروات. (B) My parents discuss with me why it is important to eat fruit and vegetables.	AS
10	(O) I sometimes give my child something to eat when they do something right, for example, when doing homework. (A) أنا أحيانًا أعطي طفلي شيئًا ليأكله عندما يفعل شيئًا صحيحًا، على سبيل المثال عند أداء الواجب المنزلي. (B) I sometimes give my child something to eat when he/she does something right, for example when doing homework.	(O) My parents sometimes give me something to eat when I do something right. For example, when doing my homework. (A) يعطيني والداي أحيانًا شيئًا لآكله عندما أفعل شيئًا صحيحًا. على سبيل المثال عند القيام بواجبي المنزلي. (B) My parents sometimes give me something to eat when I do something right. For example, when doing my homework.	CC
11	(O) I consciously eat vegetables or fruit when my child is around. (A) انا أتناول الخضار أو الفاكهة بوعي عندما يكون طفلي بالقرب مني, مثل تناولي السلطة الطازجة أو الفواكه الموسمية في وجبة العشاء. (B) I consciously eat vegetables or fruits when my child is around, such as having fresh salad or seasonal fruits during dinner.	(O) My parents consciously eat vegetables or fruit when I am around. (A) والداي يأكلان الخضار أو الفاكهة بوعي عندما أكون في الجوار ، مثل تناولهما السلطة الطازجة أو الفواكه الموسمية في وجبة العشاء. (B) My parents consciously eat vegetables or fruits when I'm around, like having fresh salad or seasonal fruits during dinner.	Mod
12	(O) I have clear rules about what my children can snack on, for example, one biscuit after school. (A) أنا لدي قواعد واضحة حول ما يمكن لأولادي تناوله كوجبة خفيفة على سبيل المثال قطعة بسكويت واحدة بعد المدرسة. (B) I have clear rules about what my children can snack on for example 1 biscuit after school.	(O) My parents have clear rules about what I can snack on, for example, one biscuit after school. (A) لدى والديّ قواعد واضحة حول ما يمكنني تناوله كوجبة خفيفة على سبيل المثال قطعة واحدة من البسكويت بعد المدرسة. (B) My parents have clear rules about what I can snack on for example 1 biscuit after school.	SS
13	(O) I make sure my child does not snack just before meals. (A) أنا أتأكد من أن طفلي لا يتناول وجبة خفيفة قبل الوجبات الرئسية مباشرة. (B) I make sure my child does not snack just before meals.	(O) My parents make sure I do not snack just before meals. (A) يتأكد والداي من أنني لا أتناول وجبة خفيفة قبل الوجبات الرئسية مباشرة. (B) My parents make sure I do not snack just before meals.	SS
14	(O) I sometimes give my child a small snack as comfort. (A) أنا أحيانًا أعطي طفلي وجبة خفيفة صغيرة كالتعتيمة. (B) I sometimes give my child a small snack, like a bite-sized treat.	(O) My parents sometimes give me a small snack as comfort. (A) يقدم لي والداي أحيانًا وجبة خفيفة صغيرة كالتعتيمة. (B) My parents sometimes give me a small snack like a bite-sized treat.	CC
15	(O) I try to consciously set a good example when it comes to eating fruit and vegetables. (A) أنا أحاول أن أكون بوعي قدوة حسنة عندما يتعلق الأمر بتناول الفاكهة والخضروات. (B) I try to consciously set a good example when it comes to eating fruit and vegetables.	(O) My parents try to consciously set a good example when it comes to eating fruit and vegetables. (A) يحاول والداي أن يكونا قدوة حسنة بوعي عندما يتعلق الأمر بتناول الفاكهة والخضروات. (B) My parents try to consciously set a good example when it comes to eating fruit and vegetables.	Mod
16	(O) I have rules about when my child is allowed to eat snacks and how much. (A) أنا لدي قواعد حول متى يسمح لطفلي بتناول الوجبات الخفيفة ومقدارها. (B) I have rules about when my child is allowed to eat snacks and how much.	(O) My parents have rules about when I am allowed to eat snacks and how much. (A) لدى والديّ قواعد بشأن الوقت الذي يُسمح لي فيه بتناول الوجبات الخفيفة ومقدارها. (B) My parents have rules about when I am allowed to eat snacks and how much.	SS
